# Profiling Sociodemographic Risk Factors and Clinical Outcomes of Women with Endometrial Cancer in Puerto Rico: The Central Role of Obesity and Obstetric Features

**DOI:** 10.1007/s40615-024-02267-8

**Published:** 2025-01-20

**Authors:** Kimberly D. García-Irizarry, María E. Rojas-Brenes, José A. Oliveras-Torres, Camila N. Ortiz-Ortiz, William D. Cress, Edna Gordián, Ricardo Gómez Martínez, Francisco J. Quintana-González, Pedro F. Escobar-Rodríguez, Teresita Muñoz-Antonia, Idhaliz Flores

**Affiliations:** 1 Puerto Rico Biobank, Ponce Health Sciences University-Moffitt Cancer Center U54 Partnerships to Advance Cancer Health Equity (PACHE), Ponce, USA; 2 Department of Molecular Oncology, Moffitt Cancer Center, Tampa, FL USA; 3 Instituto Gineco-Oncológico, San Juan, PR USA; 4 Department of Basic Sciences, Ponce Health Sciences University, Ponce, PR 00732 USA; 5 Department of Obstetrics and Gynecology, Ponce Health Sciences University, Ponce, PR USA

**Keywords:** Endometrial cancer, Risk factors, Hispanic/Latina, Epidemiology, Obesity

## Abstract

**Introduction:**

Incidence of endometrial cancer (EC) in Hispanic/Latina (H/L) women are higher compared to other race/ethnicities in the United States. EC is the third most common cancer and the fourth cause of cancer-related deaths in Puerto Rican women, yet demographic and clinical information is limited. High rates of EC risk factors such as obesity, diabetes mellitus type 2 (DM2) and hypertension (HTN) have been documented in the Puerto Rican population.

**Objective:**

To describe the demographic, clinical history, lifestyle, obstetrical-gynecological, pathologic, and molecular profiles of women with EC predominantly from Southern/Central Puerto Rico.

**Methods:**

We conducted a retrospective secondary analysis of data abstracted from the Puerto Rico Central Cancer Registry (PRCCR), self-administered questionnaires and medical records of EC cases. Descriptive statistics were conducted using SPSS V28 and RStudio.

**Results:**

We identified 105 EC cases aged 28–82. The major risk factors were BMI ≥ 30 (72%), HTN (33%), and DM2 (20%). Endometrioid adenocarcinoma was the main histological tumor type (80%), of which 74% were Type I. Obesity and nulliparity were associated with younger age at diagnosis. Older age at diagnosis (> 65 y/o) was associated with more advanced disease.

**Conclusions:**

This study defined the clinical-demographic profile of women with EC from Puerto Rico and identified risks factors that are associated with younger or older age at diagnosis.

**Impact:**

Profiling the risk factors for EC may help improve diagnostic accuracy and clinical management and result in better outcomes for this under-served, under-researched cancer patient population.

**Supplementary Information:**

The online version contains supplementary material available at 10.1007/s40615-024-02267-8.

## Introduction

Endometrial cancer (EC) is the fourth most common cancer overall and the sixth cause of cancer-related deaths in women in the United States (US) [1]. EC is the second most common cancer and the third leading cause of death due to gynecological cancer among women worldwide [2]. For the year 2023, it was estimated that there will be about 66,200 new cases of EC and around 13,030 deaths in the US [3]. The most common symptom of EC is postmenopausal bleeding, but patients with advanced disease may present with pelvic pain, abdominal distention and pain, early satiety, and changes in bladder or bowel function [4]. Standard EC management is hysterectomy with bilateral salpingo-oophorectomy with surgical staging [5] that usually involves removal of the pelvic lymph nodes. In young premenopausal women, ovarian preservation can be considered to avoid early surgical menopause. The goal is to remove the primary tumor with all visible disease and determine if adjuvant treatment is needed. Patients with early-stage disease have a very good prognosis. Most EC recurrences (symptomatic or asymptomatic) will occur within the first two years after primary treatment, however, detection of relapse in asymptomatic patients is low [6].

EC can be divided in estrogen dependent non-aggressive (Type I) and estrogen independent (Type II) histologic types. Type I includes low grade endometrioid adenocarcinoma (grade 1 and grade 2) and tends to be more common with a good prognosis. Type II includes high grade endometrioid adenocarcinoma (grade 3), serous carcinoma, clear cell carcinoma, mixed carcinoma, undifferentiated carcinoma, carcinosarcoma, other unusual types (e.g. mesonephric-like) and gastrointestinal mucinous type carcinomas. Type II EC has a poorer prognosis with a more fatal outcome [5].

The risk factors for EC are obesity, hypertension (HTN), and diabetes mellitus type 2 (DM2) which are modifiable [7–9]; non-modifiable risk factors include Lynch Syndrome (LS) and other hereditary cancer syndromes. Women with obesity class I (defined as BMI > 30 and < 35 kg/m^2^) have more than 2.6-fold risk of developing EC [9], most likely low-grade EC [10–12]. Furthermore, women with DM2 have more than 72% increased risk of developing EC [13]. These risk factors have an increased prevalence among Hispanic/Latino (H/L) in comparison to Non-Hispanic Whites (NHW) [14]. In Puerto Rican women, obesity, HTN and DM2 are highly prevalent thus increasing their lifetime risk for EC [15, 16].

In the US, NHW, Non-Hispanic Blacks (NHB), and H/L have similar incidence rates of EC with higher mortality rates in NHB [17]. Moreover, EC has the third highest cancer incidence and is the fourth cause of cancer-related deaths in Puerto Rican women [1]. From 1969 to 1971, women living in Puerto Rico had a lower incidence of EC in comparison to women of all races in the US [18]. However, the incidence rate of EC in Puerto Rico has significantly increased during the last several years. The mortality of EC from 1999 to 2003 was higher in Puerto Rico in comparison to US mainland H/L [19]. More recently, the age-adjusted (20 to 64 years of age) incidence rate of EC from 2000 to 2018 remains the highest in Puerto Rican women in comparison to H/L, NHB, NHW, and the US overall. Also, the mortality rate was higher in Puerto Rican women with ages from 35 to 49 in comparison to other racial and ethnic groups; however, NHB continue to have the highest mortality rate overall [20].

Despite the alarming increasing trend of EC and the high prevalence of risk factors in Puerto Rican women, there is still limited information. This study aims to analyze recent data on demographic, clinical, lifestyle, obstetrical-gynecological (ob-gyn), pathologic, and molecular profiles of Puerto Rican women with EC who donated tissues to the Puerto Rico Biobank (PRBB), a cancer-focused tissue biorepository serving Southern Puerto Rico. The population in this region is socio-economically disadvantaged and understudied, which provides an opportunity to better understand health disparities in cancer treatment [21]. Our findings will improve our understanding of how EC risk factors in Puerto Rican women influence clinical management and outcomes.

## Materials and Methods

### Data Sources

This is a retrospective secondary data analysis from the PRBB database, the tissue repository of the Ponce Health Sciences University (PHSU)-Moffitt Cancer Partnership, sponsored by the National Cancer Institute (NCI) of the National Institutes of Health (NIH). We evaluated 105 cases of primary EC (ICD-O-3), specifically sites C54.1 (malignant neoplasm of endometrium) and C54.9 (malignant neoplasm of corpus uteri). These cases involved individuals who consented to donate tissues and data to the PRBB between the years 2010 to 2019. Puerto Rican women aged between 21 and 89, most of whom live in Southern/Central Puerto Rico. Data was abstracted from the PRBB Biospecimen Management System (BMS), the Puerto Rico Central Cancer Registry (PRCCR) database, self-reported questionnaires, pathology reports, and medical records. The PRCCR is the island's epidemiological cancer surveillance system, responsible for collecting, analyzing, and disseminating cancer information, such as demographics and clinical data (www.rcpr.org).

### Study Variables

Demographic characteristics included age, age at diagnosis, educational level, marital status, employment status, zip code, and vital status. Lifestyle characteristics included diet (e.g., regular, diabetic, low salt, high in fiber, low in carbs, unknown). Regular diet was defined as having no dietary restrictions. Physical activity was assessed based on activities undertaken during the previous week. Smoking behavior (current, past smoker, nonsmoker, secondhand smoke exposure). Subjects who had consumed at least 100 cigarettes in their lifetime were considered smokers. Alcohol consumption (current, past alcohol use, none) and non-steroidal anti-inflammatory drugs (NSAID) exposure (< 10 years, ≥ 10 years) were also recorded. Information on BMI was abstracted from PRBB database and medical records. BMI was categorized as non-obese (BMI < 30 kg/m^2^) and obese (BMI ≥ 30.0 kg/m^2^).

Ob-gyn data included age at menarche, menstrual cycle characteristics, dysmenorrhea, number of pregnancies, age of first pregnancy, breastfeeding, menopausal status, oral contraceptive (OC) use, hormone replacement therapy (HRT), frequency of gynecologic visits, co-morbidities, risk factors (such as BMI and, history of DM2 and HTN) and family history of cancer. Regular menstrual cycle was defined as menstrual flow every 24 to 38 days for up to 8 days (ACOG, 2020).

Pathological characteristics of cases included histological type of the tumor (Type I and Type II), FIGO grade (1, 2, 3, low grade and high grade), FIGO stage (I, II, III and IV) and treatment (surgery and combined therapy). Molecular characteristics were abstracted from pathology reports, including protein expression assays for MLH1, MSH2, MSH6, and PMS2.

### Statistical Analysis

Descriptive analyses were performed for demographic, ob-gyn, clinical, lifestyle and molecular data. Numerical variables were analyzed by means, frequencies, and percentages. Significant differences were assessed using Chi-Square and Fisher’s Exact tests for categorical variables; the t-test was used to assess group differences for continuous variables. A two-sided *p*-value of ≤ 0.05 was considered statistically significant. The Mann–Whitney U Test was used to assess the association between age at diagnosis and risk factors. All statistical analyses were performed using SPSS version 28 (IBM Inc., Armonk, NY), R (R Core Team, 2020) [22], and RStudio (RStudio Team, 2020) [23].

## Results

### Demographic Characteristics

A total of 105 cases of women with EC were included in the study. Table 1 presents demographics, lifestyle, and clinical characteristics. The mean age was 57 years (SD ± 12), with a range of 28–82. More than half had only up to high school education (58.2%) and were unemployed (58%) and 50% were married. The majority (85%) of cases were from the Southern/Central regions of Puerto Rico. Most participants (90%) were alive at the time of data abstraction. Most participants identified as Hispanic/Latino (100%) and White (65%).

**Table 1:** Demographic, lifestyle, and clinical characteristics

Characteristics	Total participants (n)	No. (%)
Age, mean (SD)	105	57.4 (12.3)
Age range (yrs.)		28—82
≤ 40		13 (12.4)
41–50		17 (16.2)
51–60		27 (25.7)
61–70		35 (33.3)
≥ 71		13 (12.4)
Education level, ≤ High school	91	53 (58.2)
Race		
White		N (65.1%)
Black		N (7%)
Preferred not to answer		N (27.9%)
Employment status, Unemployed	105	61 (58.1)
Marital Status	96	
Single		23 (24.0)
Married		48 (50.0)
Divorced		9 (9.4)
Widowed		7 (7.3)
Live with partner		9 (8.6)
BMI, mean (SD)	90	33.4 (7.2)
BMI range		15.8–52.3
BMI groups		
< 30		25 (27.8)
≥ 30		65 (72.2)
Smoking Status	97	
Current or past smoker		10 (10.3)
Nonsmoker		47 (48.5)
Secondhand exposure		40 (41.2)
Alcohol Use	97	
Current		16 (16.5)
Past alcohol use		19 (19.6)
Non-alcohol use		62 (63.9)
NSAID exposure	36	
< 10 years		15 (14.3)
≥ 10 years		21 (20.0)
Comorbidities	105	
Diabetes Mellitus Type II		21 (20.0)
Hypertension		35 (33.3)
Hyperlipidemia		17 (16.2)
Thyroid Disease		11 (10.5)
Vital Status	93	
Alive		84 (90.3)
Dead		9 (9.7)
Diet	88	
Regular diet		74 (84.1)
Diabetic diet		10 (11.4)
Low Salt diet		4 (4.5)
High Fiber diet		0 (0)
Low Carbs diet		0 (0)
Physical activity	81	
Yes		30 (37.0)
No		51 (63.0)

### Clinical History

Comorbidities evaluated in our study included chronic conditions known to be risk factors for EC. One third (33%) of the study cohort self-reported HTN, 20% DM2, 16% hyperlipidemia, and 11% thyroid disease (Table 1).

### Lifestyle Factors

Most participants (90%) were nonsmokers, while 41% were exposed to secondhand smoke; 64% reported no alcohol consumption. Participants who were obese represented 72% of the cohort (Table 1). Most patients (80%) were on a regular diet and 63% reported no physical activity.

### Obstetrical-Gynecological (ob-gyn) History

The mean age at menarche was 12 years (SD ± 1.7). Approximately one third (26%) of the participants reported irregular menses, 52% reported intermenstrual bleeding and 70% reported dysmenorrhea. Nulliparous women represented 22% of the cohort. Ever pregnant cases had a higher mean age (61 y/o) at diagnosis than nulliparous (47 y/o) (*p* < 0.001). The mean age for initial pregnancy was 21 y/o. Only one third (33%) reported breastfeeding. About half (55%) of the participants had experienced menopause, with a mean age of menopause onset at 49 y/o (SD ± 6.7). Less than a half (46%) reported ever using hormonal contraception; of those the majority (90%) had used OC in their lifetime (Table 2).

**Table 2:** Obstetrical-gynecological history

Characteristic	Total participants (n)	No. (%)
Irregular menstrual cycle	80	21 (26.3)
Intermenstrual bleeding	77	40 (51.9)
Dysmenorrhea	79	55 (69.6)
Endometriosis	82	15 (18.2)
Parity	93	
0		20 (21.5)
1		7 (7.5)
2		19 (20.4)
≥ 3		47 (50.5)
Miscarriages, yes	105	19 (18.1)
Age (yrs.) at menarche, mean (SD)	105	12 (1.7)
Age (yrs.) at menarche, range	105	8–16
Menopause, yes	58	58 (55.2)
Age at menopause (yrs.), mean (SD)	51	49 (6.7)
Age at menopause range		32—62
< 55 y/o (early)		39 (76.5)
≥ 55 y/o (late)		12 (23.5)
Pre-menopausal	87	27 (31.0)
Ever use hormonal contraception, yes	84	39 (46.4)
Ever use of oral contraceptives, yes	30	27 (90)
Ever use of hormone replacement therapy	84	4 (4.8)
Age at 1st pregnancy, mean (SD)		21 (3.5)
Age at 1st pregnancy, range (yrs.)		15–30
Breastfeeding, yes	85	28 (32.9)

### Endometrial Cancer (EC) Pathologic Characteristics

About half (53%) of the participants had a family history of cancer, and 19% had a family history of EC (Table 3). The majority (86%) presented with early FIGO stages of EC (1A, 1B, 2) at the time of diagnosis, and the majority (86%) were of low FIGO grade. The most common histological type in our cohort was endometrioid adenocarcinoma (80%). A high proportion of cases (74%) presented Type I (estrogen-dependent) tumors. Regarding treatment, almost all patients (99%) underwent a hysterectomy without adjuvant therapy (radiotherapy or chemotherapy).

**Table 3:** Endometrial cancer characteristics

Characteristic	Total participants (n)	No. (%)
Family history of cancer	89	47 (52.8)
Family history of endometrial cancer	86	16 (18.6)
FIGO Stage	93	
I		74 (79.6)
II		9 (9.7)
III		8 (8.6)
IV		2 (2.2)
FIGO Grade	92	
1		56 (60.9)
2		23 (25.0)
3		13 (12.4)
Low (1–2)		79 (85.9)
High (3)		13 (14.1)
Endometrial cancer histology type	105	
Endometrioid adenocarcinoma		84 (80.0)
Other (serous, carcinoma, mullerian mixed, clear cell, sarcoma, squamous cell)		21 (20.0)
Endometrial cancer type	103	
Type I (estrogen-dependent)		78 (74.3)
Type II		19 (18.1)
NOS		6 (5.7)
Treatment	105	
Hysterectomy only		104 (99.0)
Hysterectomy + Radiotherapy		5 (4.8)
Hysterectomy + Chemotherapy		3 (2.9)

Only seven tissue samples were evaluated at the molecular level in our study cohort. The most common molecular tests done were MLH1, MSH2, MSH6, and PMS2, with all cases presenting positive MSH6 expression (Supplemental Tables 1 and 2).

### Association Analysis of Variables of Interest

We next analyzed potential associations between study variables of interest (age at diagnosis, family history, HTN, DM2, BMI, breastfeeding and ever been pregnant) with histology type (Endometrioid vs. Non-endometrioid), cancer type (Type I vs. Type II), FIGO Grade 1–3 and FIGO Stage 1–4.

Using Fisher Exact Test and Pearson Chi Square, we found no significant associations between having Endometrioid vs. Non-endometrioid cancer with age at diagnosis, HTN, DM2, BMI, family history, or ever being pregnant. Women with endometrioid cancer had a higher proportion of BMI ≥ 30 (84%) compared with non-endometrioid cancer; however, this difference did not reach statistical significance (*p* = 0.084) (Table 4). A higher proportion of those with no history of breastfeeding had endometrioid tumor types (46 cases, 68.7%). However, no significant association was observed between tumor type (endometrioid vs. non-endometrioid) and breastfeeding history (Yes/No) (χ^2^ = 0.590; *p* = 0.566). We observed a significant association between the FIGO Stage and Grade, and the histology of the diagnosis (Endometrioid vs. Non-endometrioid) (χ^2^ = 9.51, *p* < 0.05; χ^2^ = 24.32). Specifically, the frequency of endometrioid cases among those with FIGO stage 1 and 2 and FIGO grade 1 and 2 was significantly higher. The effect size, measured by Cramer's V, was moderate (V = 0.37), indicating a meaningful relationship between FIGO stage and the risk of endometroid cancer.

**Table 4:** Association of variables of interest with histology type (Endometrioid vs. Non-endometrioid)

Variables	Total Patients					*P*-value
		Endometroid		Non-Endometroid		
		*n*	(%)	*n*	(%)	
Age at diagnosis	105					0.171^a^
≤ 40		13	100	0	0	
41–50		14	82.4	3	17.6	
51–60		22	81.5	5	18.5	
61–70		27	79.4	7	20.6	
≥ 71		8	61.5	5	38.5	
FIGO Stage	93					0.012^a^
I		64	86.5	10	13.5	
II		8	88.9	1	11.1	
III		5	62.5	3	37.5	
IV		0	0	2	100	
FIGO Grade	92					< 0.001^a^
1		52	92.9	4	7.1	
2		22	95.7	1	4.3	
3		4	30.8	9	69.2	
Hypertension	104					0.5974^b^
Yes		26	76.5	8	23.5	
No		58	82.9	12	17.1	
Diabetes mellitus	104					0.758^b^
Yes		16	76.2	5	23.8	
No		68	81.9	15	18.1	
BMI	89					0.139^b^
< 30		17	68.0	8	32.0	
≥ 30						
Family History	89					0.200^b^
Yes		40	85.1	7	14.9	
No		31	73.8	11	26.2	
Ever pregnant	95					1.000^b^
Yes		60	80.0	15	20.0	
No		16	80.0	4	20.0	

^a^ Fisher-Freeman-Halton Exact Test, due to expected values under five

^b^ Pearson Chi Square

Table 5 shows the associations between FIGO Stage and variables of interest. The *p*-value for the association between FIGO stage and BMI (< 30; ≥ 30) in women with EC was (χ^2^ = 7.28, *p* < 0.034), indicating that women with earlier FIGO stage were associated with obesity. There were no statistically significant associations between FIGO stage and age at diagnosis, HTN, DM2, family history, breastfeeding and ever pregnant. Analysis of association between normal BMI vs. overweight vs. obese and FIGO Stage was not statistically significant (χ^2^ = 9.701, *p* = 0.138. Table 5 shows the associations between FIGO Grade and variables of interest. We observed that lower FIGO grade was associated with ever being pregnant (*p* = 0.006), suggesting a protective effect from pregnancy. FIGO Grade was not significantly associated with age at diagnosis, HTN, DM2, BMI, and family history.

**Table 5:** Association of variables of interest with FIGO Stage (I, II, III, IV) and FIGO Grade (1, 2, 3)

Variables	Total patients	FIGO Stage								*P*-value
		I		II		III		IV		
		n	(%)	n	(%)	n	(%)	n	(%)	
Age at diagnosis	93									
≤ 40		10	100	0	0	0	0	0	0	0.187^a^
41–50		10	71.4	1	7.1	3	21.4	0	0	
51–60		21	87.5	1	4.2	2	8.3	0	0	
61–70		25	78.1	5	15.615.4	2	6.3	0	0	
≥ 71		18	61.5	2		1	7.7	2	15.4	
Hypertension	93									0.497^a^
Yes		26	81.3	4	12.5	1	3.1	1	3.1	
No		48	78.7	5	8.2	7	11.5	1	1.6	
Diabetes mellitus	93									0.115^a^
Yes		15	78.9	0	0	3	15.8	1	5.3	
No		59	78.7	9	12.2	5	6.8	1	1.4	
BMI	80									0.034^a^
< 30		17	85.0	0	0	1	5.0	2	10.0	
≥ 30		47	78.3	8	13.3	5	8.3	0	0	
Family History	80									0.226^a^
Yes		38	86.4	4	9.1	1	2.3	1	2.3	
No		28	77.8	2	5.6	5	13.9	1	2.8	
Ever pregnant	86									0.902^a^
Yes		57	81.4	6	8.6	5	7.1	2	2.9	
No		13	81.3	1	6.3	2	12.5	0	0	

Variables	Total patients	FIGO Grade						*P*-value		
		1		2		3				
		n	(%)	n	(%)	n	(%)			
Age at diagnosis	92							0.277^a^		
≤ 40		9	90	1	10.0	0	0			
41–50		10	66.7	3	20.0	2	13.3			
51–60		17	68.0	6	24.0	2	8.0			
61–70		16	53.3	9	30.0	5	16.7			
≥ 71		4	33.3	4	33.3	4	33.3			
Hypertension	93							0.168^a^		
Yes		15	51.7	11	37.9	3	10.3			
No		41	65.1	12	19.0	10	15.9			
Diabetes mellitus	92							0.804^a^		
Yes		10	55.6	5	27.8	3	16.7			
No		46	62.2	18	24.3	10	13.5			
BMI	78							0.066^a^		
< 30		9	45.0	5	25.0	6	30.0			
≥ 30		39	67.2	14	24.1	5	8.6			
Family History	78							0.224^a^		
Yes		27	62.8	8	18.6	8	18.6			
No		20	57.1	12	34.3	3	8.6			
Ever pregnant	84							**0.006**^**a**^		
Yes		45	65.2	14	20.3	10	14.5			
No		4	26.7	9	60.0	2	13.3			

^a^ Fisher-Freeman-Halton Exact Test, due to expected values under five

No significant associations were found between having Type I vs. Type II EC with age at diagnosis, FIGO grade and stage, BMI, HTN, DM2, family history or ever being pregnant, using the Fisher Exact Test (Data not shown).

An Independent Sample T-test was performed to evaluate associations between age at diagnosis and variables of interest. Age at diagnosis was significantly higher in cases with high FIGO grade (*p* = 0.031), HTN (*p* = 0.006) and ever being pregnant (*p* < 0.001) (Table 6**)**. In contrast, women who reported having dysmenorrhea (*p* = 0.024) had a significantly younger age at diagnosis. There were no statistical differences between the age at diagnosis and DM2, BMI or family history.

**Table 6:** Association of variables of interest with age at diagnosis

Variables	Age at Diagnosis			
	Mean	N	T-test	*P*-value
BMI			1.342	0.183^a^
< 30	60.36	25		
≥ 30	56.63	65		
FIGO Grade			−2.189	**0.031**^**a**^
Low (Grade I-II)	56.81	79		
High (Grade III)	64.54	13		
Hypertension			2.784	**0.006**^**a**^
Yes	61.94	35		
No	55.06	70		
Diabetes Mellitus			0.643	0.521^a^
Yes	58.90	21		
No	56.96	84		
Dysmenorrhea			−1.851	**0.068**^**a**^
Yes	55.20	55		
No	60.50	24		
Family History			−0.147	0.884^a^
Yes	56.94	47		
No	57.31	42		
Ever pregnant			5.085	** < 0.001**^**a**^
Yes	60.55	76		
No	47.05	20		

^a^ Independent Sample T-test

## Discussion

EC is one of the most prevalent gynecologic malignancies in Puerto Rico; however, the clinico-demographic profile of patients from this representative H/L group remains limited. The present study provides updated information on clinical profiles and the prevalence of associated risk factors focusing on patients from Southern/Central regions of Puerto Rico. In this region, located 74 miles from the metropolitan area of San Juan, cancer care is de-centralized and there are documented disparities related to their lower socioeconomic status, low health literacy rates, and limited access to care [21]. We present the results from a secondary analysis of data obtained from the PRBB; a cancer-focused tissue procurement facility located in the Ponce Health Region of Puerto Rico. Our assessment validated the high prevalence of the most well-known risk factors and their association with EC subtypes and FIGO Stage and Grade, while also uncovering new associations.

In comparison to other studies conducted on the Puerto Rican population [19, 20, 24], our cohort had greater percentage of patients diagnosed after 60 years of age. Only 3.8% (*n* = 4) were diagnosed at 30 years of age or younger, similar to the observations by Ortiz et al. and Rosario-Santos et al. [19, 20]. Importantly, Charneco et al.’s study reported 19% unemployment compared to 58% in our study [24]​​. This substantial difference in employment status highlights a clear distinction in the socio-economic backgrounds between Puerto Rican patients based on geographical residence or point-of-care (metropolitan area vs. other island regions), in accord with Census data documenting a higher rate of poverty in Southern/Central Puerto Rico [25]​. This is important as low socioeconomic status has been associated with worse cancer outcomes [26, 27].

More than half (69%) of our cohort reported being postmenopausal, with a mean age of 49 y/o of menopause onset, like what has been reported previously in Puerto Rico [24]. ​This is consistent with what has been reported at the national level, where EC is prevalent in postmenopausal women [17]. The mean age at menopause onset is in accord with what has been reported for H/L and NHB women, and lower than that of NHW women in the US (52 y/o), an interesting finding that warrants further research to understand its implications for cancer risk [28]. In addition to menopausal status, the number of pregnancies, use of OC, HRT, history of breastfeeding and age at menarche were analyzed. Nulliparity is a well-established risk factor for EC [29], which in our cohort was 21%, similar to the findings by Charneco et al. [24]. Also, we observed that patients who were ever pregnant (at least one child) had a higher mean age at diagnosis (61 y/o) in comparison to the nulliparous group (average age at diagnosis 47 y/o) (*p* < 0.001). The association between nulliparity and early cancer development is unclear, as there may be many confounding factors including obesity which is also related to infertility [30, 31]. Thus, our study provides additional evidence for the role of nulliparity in increasing the risk of younger age at diagnosis with EC. Breastfeeding has been shown to be a protective factor for breast and ovarian cancer, however, we did not find significant associations between FIGO Stage nor endometrioid vs. non-endometrioid tumor type (although a higher proportion of those with no history of breastfeeding had endometrioid tumor types (46 cases, 68.7%) and breastfeeding history. Additional studies with a larger sample size would be required to further explore this potential association, especially relevant to endometrial cancer as lactation has well known benefits to metabolic health related to hypertensive and diabetic states [32].

The most common risk factors among our subjects were obesity (72%), HTN (33%), and DM2 (20%); these values are comparable to previously reported data from Puerto Rican patients [24]. We did not find a significant association between DM2 and HTN with EC. Cook et al. [14] found that obesity, DM2 and HTN were more common among H/L women vs NHW; however, DM2 was not significantly associated with EC and did not affect mortality [14]. A strong association between obesity and a diagnosis of endometrioid-type EC has been established [33]. Our study validates previous observations of associations between higher BMI and younger age at diagnosis [8]. For example, BMI > 40 kg/m^2^ has been associated with younger age at diagnosis of endometrioid EC but not to higher-grade, aggressive EC (non-endometrioid) [8]. In our cohort, about three-fourths (72%) of participants had a BMI ≥ 30 kg/m^2^. Our findings are comparable to other studies of Puerto Rican patients [24] showing four-fold increased risk of having EC with BMI ≥ 30 kg/m^2^. A possible reason for higher BMI values in our cohort is that most of the subjects reported no restrictions in diet and many reported low to no physical activity.

Although the pathological characteristics of our cohort are like those of Charneco et al.’s [24], there is a significant difference in the proportion of selected treatments for EC. Most of our cohort (99%) had only surgery (hysterectomy with salpingo-oophorectomy), compared to 68% reported in the previously mentioned study. Additionally, this metropolitan area cohort was treated with surgery and radiotherapy in 18% of cases, a higher proportion compared to 5% in our cohort. This was probably due to lower proportion of cases with FIGO stage II in our study (10% vs 22%), respectively. However, we observed that older patients in our cohort had a higher likelihood to present with a high FIGO grade and have a higher risk of a more aggressive tumor (*p* = 0.031). These findings suggest that older patients in our cohort either had a delayed diagnosis of EC or the cancer started later in life. This is clinically impactful as it is well-known that older patients have a greater risk of complications and mortality from EC [33].

Some of the limitations of the study include the relatively small sample size, and a retrospective, descriptive analysis that cannot assess causality. There was limited information about family history of cancer and of hormone replacement therapy. In addition, limited data were found regarding molecular testing, with 7% of participants only having had IHC testing for MMR proteins MLH1/ MSH2 / MSH6/ PMS2, which is necessary for the diagnosis for LS. Four of the participants (57%) had loss of at least one expression of MMR proteins, and three of them were younger than 50 years old, and only one reported family history of EC. Therefore, our study revealed a health disparity within our cohort due to the limitations in identifying potential cases of LS.

In summary, this study identified a high prevalence of obesity, HTN and DM2 associated with EC and evaluated their association with various EC subtypes, including endometrioid vs. non-endometrioid; estrogen-dependent (Type I) vs. non-estrogen dependent (Type II), FIGO stage, and grade. Notably, we found that older patients (≥ 65y/o) were more likely to present with advanced disease, and validated previously documented associations between obesity and diagnosis of EC. This study describes for the first time the clinico-demographic profile of women with EC from Southern/Central regions of Puerto Rico, revealing well-established and emerging associations between younger age at diagnosis and obesity and nulliparity. This profile will facilitate recognition of risk factors and diagnostic features and promote prompt and improved clinical management of this understudied cancer patient population.

## Supplementary Information

Below is the link to the electronic supplementary material.

Supplementary file1 (DOCX 13.2 KB)

## Data Availability

De-identified data from this study are available upon reasonable request to the contact author (iflores@psm.edu).
