# Supplementary material for: Profiling Sociodemographic Risk Factors and Clinical Outcomes of Women with Endometrial Cancer in Puerto Rico: The Central Role of Obesity and Obstetric Features
Source: J Racial Ethn Health Disparities. 2025 Jan 20;13(1):552–61. doi: 10.1007/s40615-024-02267-8 (PMC12795853; doi:10.1007/s40615-024-02267-8)
Supplement: Supplementary file 1 — Supplementary file1 (DOCX 13.2 KB) [file 40615_2024_2267_MOESM1_ESM.docx]

**SUPPLEMENTAL TABLES**

**Supplemental Table 1: Protein expression of MLH1, MSH2, MSH6, and PMS2 results**

| Protein | Presence | Absence |
| --- | --- | --- |
| MLH1 | 4 | 3 |
| MSH2 | 5 | 2 |
| MSH6 | 7 | 0 |
| PMS2 | 5 | 2 |
|  |  |  |

**Supplemental Table 2:** Results from the seven subjects analyzed for MMR protein expression are shown.

| Case | MLH1 nuclear expression | MSH2 nuclear expression | MSH6 nuclear expression | PMS2 nuclear expression | Family history | BMI | Age at diagnosis |
| --- | --- | --- | --- | --- | --- | --- | --- |
| 1 | Absence | Present | Present | Absence | no | 26.9 | 39 |
| 2 | Present | Present | Present | Present | no |  | 49 |
| 3 | Present | Present | Present | Present | no | 37.6 | 62 |
| 4 | Present | Present | Present | Present | no | 31 | 65 |
| 5 | Absence | Present | Present | Absence | no |  | 75 |
| 6 | Absence | Absence | Present | Present | yes | 29.9 | 49 |
| 7 | Present | Absence | Present | Present | no |  | 29 |
| Absent protein | 3 | 2 | 0 | 2 |  |  |  |
